# Electron, hole, and energy transfer dynamics in non-fullerene small-molecule acceptors†

**DOI:** 10.1039/d4sc04072d

**Published:** 2024-09-18

**Authors:** Guangliu Ran, Bo Zhuang, Jiulong Huang, Hao Lu, Yahui Liu, Zhishan Bo, Feng Gai, Wenkai Zhang

**Affiliations:** a School of Physics and Astronomy, Applied Optics Beijing Area Major Laboratory, Center for Advanced Quantum Studies, Beijing Normal University, Beijing 100875 China wkzhang@bnu.edu.cn; b Beijing National Laboratory for Molecular Sciences, College of Chemistry and Molecular Engineering, Peking University Beijing 100871 China fgai@pku.edu.cn; c College of Materials Science and Engineering, Qingdao University Qingdao Shandong 266071 China; d College of Textiles and Clothing, State Key Laboratory of Bio-fibers and Eco-textiles, Qingdao University Qingdao Shandong 266071 China; e Key Laboratory of Multiscale Spin Physics, Ministry of Education, Beijing Normal University Beijing 100875 China

## Abstract

When photoexcited, an organic photovoltaic (OPV) donor/acceptor (D/A) blend is expected to undergo charge separation (CS) through three channels: electron transfer, hole transfer, and energy transfer-induced electron/hole transfer. However, previous spectroscopic studies on various blends based on non-fullerene acceptors (NFAs) have not been able to directly characterize the dynamics of these processes, due to spectral overlap of the involved intermediate species. Herein, we study the excited-state dynamics of D/A blends composed of PBDB-T (D) and a L-series NFA (L4 or L5) and show that the species responsible for these processes in the PBDB-T/L4 blend can be spectroscopically identified, allowing us to disentangle their dynamics. Moreover, we confirm the occurrence of photoinduced CS in neat L4 and L5 films, providing direct evidence that CS can occur under nearly zero driving force in OPV systems. Further density functional theory calculations suggest that specific molecular packing patterns may play an important role in facilitating CS.

## Abstract 2

Creation of free charge carriers or charge separation (CS) is a key event in organic photovoltaic (OPV) devices. We report the dynamics and mechanisms of CS in OPV thin films and provide direct evidence that CS can occur when the underlying driving force is nearly zero.

## Graphical abstract

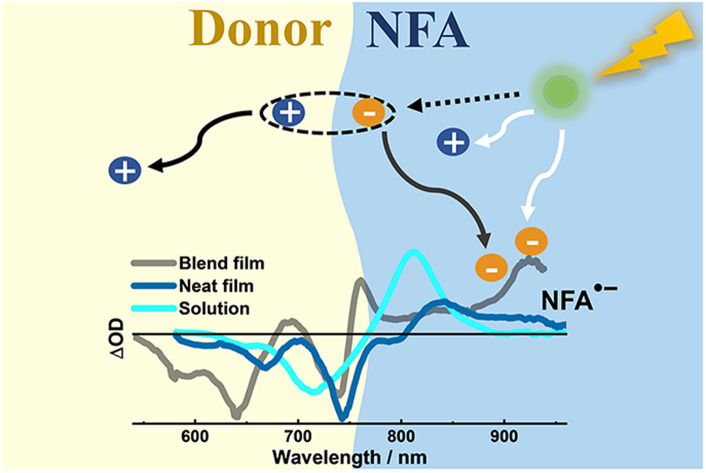

## Introduction

Solar energy conversion using organic photovoltaic (OPV) devices has emerged as a promising avenue to address the growing global demand for sustainable and renewable energy sources.^1–4^ In particular, great efforts have been made to develop non-fullerene acceptors (NFAs) that can significantly increase the power conversion efficiency (PCE) of OPV devices.^5–10^ In comparison to fullerene-based acceptors, NFAs show great potential in enhancing electron transport, reducing voltage loss, and facilitating charge separation (CS).^11,12^ Currently, most high-performance NFAs adopt a symmetric architecture that has a fused-ring conjugated core with strongly electron-deficient terminal groups attached to its two sides, and the modularity of such a design has allowed various chemical modifications to tune key molecular determinants of PCE, such as energy levels, the absorption spectrum, solubility, and bulk morphology.^7,13–15^ For example, the Y-series NFA Y6 (Fig. S1†) employs a ladder-type electron-deficient-core-based central fused ring (dithienothiophen[3.2-*b*]-pyrrolobenzothiadiazole) with 2-(5,6-difluoro-3-oxo-2,3-dihydro-1*H*-inden-1-ylidene)malononitrile as flanking groups to enhance visible-light absorption and promote intermolecular interactions. When it is blended with the electron donor PM6, the solar cell delivers an efficiency of 15.7%.^16,17^ While the performance of OPV devices is continuously being improved and previous studies have provided significant insights into how such polymeric blends can achieve high PCEs,^18–22^ a mechanistic and predictive understanding of the mechanism of a key process in photocurrent generation, *i.e.*, conversion of excitons to free charges, is still lacking.

In NFA-based heterojunction OPVs, electron–hole separation is believed to occur on an ultrafast timescale and with a near 100% yield, even under conditions where the underlying driving force (or energy offset) is small.^11,23^ Based on various time-resolved spectroscopic studies, several mechanisms have been proposed to rationalize the high efficiency of CS in such heterojunctions.^18–20,24–28^ For example, Wang *et al.*^18^ reported that an intra-moiety excited state (i-Ex) species with an absorption feature at *ca.* 1550 nm may act as an intermediate state for the hole transfer (HT) channel in the PM6/Y6 blend. This dimeric excited state that first forms at the same Y6 moiety away from the interfaces was suggested to rationalize the highly efficient HT in PM6/Y6 blends despite a small HOMO offset between the donor and acceptor at the interface.^18,29^ Price *et al.* also demonstrated the occurrence of barrier-free CS in pure Y6 films.^20^ However, it still remains elusive whether such an intra-moiety CS (or quasi-CS) state is generally involved in the functional processes of different NFAs with similar intra-molecular electron pulling and pushing components. Other studies have provided additional insights into this issue. For instance, Song *et al.*^26^ found that bound polaron pairs generated within NFA domains play a dominant role in efficient CS and HT with near zero driving force. Zhang *et al.*^30^ showed that polymerization of NFAs can serve as an effective strategy to increase free carrier concentration. Niu *et al.*^31^ reported that the HT induced CS is mediated by weakly bound exciton dissociation generated among NFA molecules rather than Frenkel exciton dissociation. However, these studies lack direct spectroscopic evidence confirming the generation of charged species (*i.e.*, the radical anion of NFAs) in NFA domains. To further address this issue, and obtain a better understanding of the CS mechanism in OPVs composed of NFAs, herein we use broadband transient absorption (TA) spectroscopy to assess the excited-state dynamics of two L-series NFAs (*i.e.*, L4 and L5), in blends with a polymeric electron donor, PBDB-T (Fig. S1†), as well as in their corresponding pristine states, and use molecular dynamics (MD) simulations, density functional theory (DFT) calculations and its time-dependent variant (TDDFT) to examine their excited-state electronic properties.

## Results and discussion

**Fig. 1:**
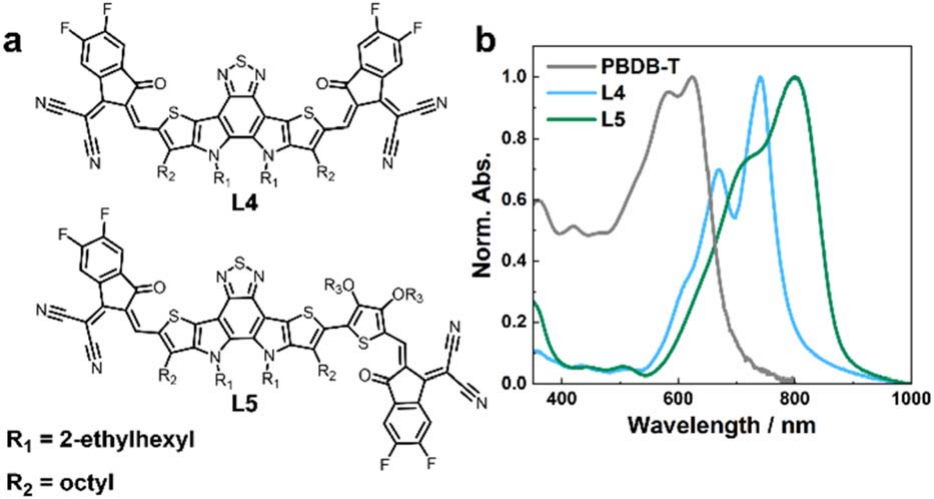
(a) Chemical structures of L4 and L5, as indicated. (b) Absorption spectra of L4, L5, and PBDB-T neat film samples, as indicated.

As shown in Fig. 1a, L4 and L5 are structurally similar to Y6 (Fig. S1†), but with a smaller core component, and in L5, thiophene alkoxy units are introduced between the core and the end groups, rendering the structure asymmetrical, to improve the solubility, phase morphology, and charge transport capability.^32^ The absorption spectra of these NFAs are well separated from that of the PBDB-T donor in both free and blend forms (Fig. 1b and S2†), making selective excitation of either the donor or acceptor in the blends possible.

**Fig. 2:**
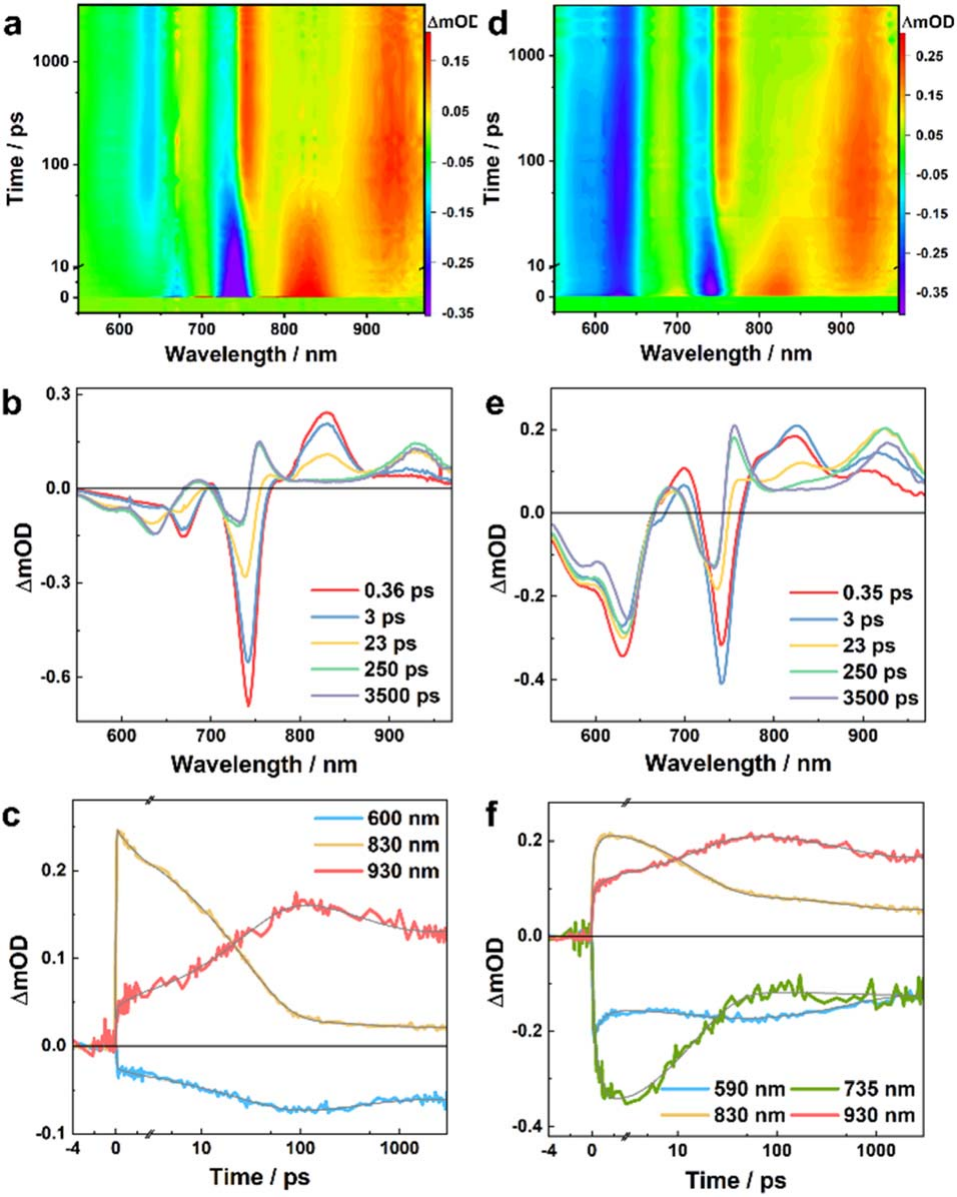
TA results of the PBDB-T/L4 blend obtained with an excitation wavelength of 800 nm (a, b and c) and 550 nm (d, e and f), respectively. (a and d) Two-dimensional false color TA map. (b and e) TA spectra at different time delays, as indicated. (c and f) TA kinetic traces at different wavelengths, as indicated, where the smooth lines represent the global fits of these curves to the kinetic function described in the main text.

**Fig. 3:**
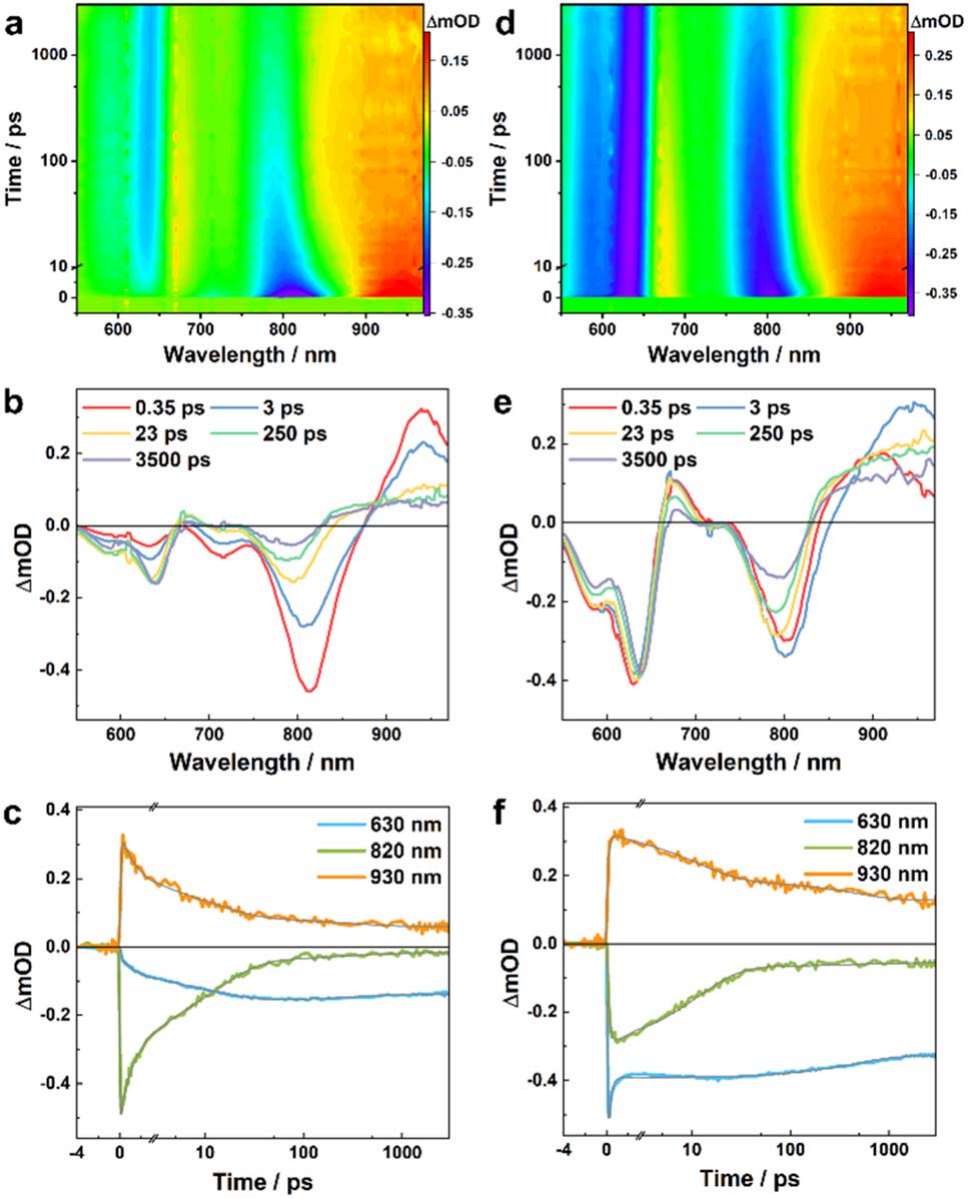
TA results of the PBDB-T/L5 blend obtained with an excitation wavelength of 800 nm (a, b and c) and 550 nm (d, e and f), respectively. (a and d) Two-dimensional false color TA map. (b and e) TA spectra at different time delays, as indicated. (c and f) TA kinetic traces at different wavelengths, as indicated, where the smooth lines represent the global fits of these curves to the kinetic function described in the main text.

To directly characterize the photoinduced HT dynamics, we first measured the TA spectra of these PBDB-T/Ln blends with a pump wavelength of 800 nm, which selectively excites the Ln in the blends. As shown in Fig. 2, at early time delays (*e.g.*, 0.4 ps) the TA spectra consist of an expected negative or ground-state bleaching (GSB) signal in the region of the steady-state absorption spectra of these NFAs (Fig. 1b) and an excited-state absorption (ESA) feature arising from the locally excited (LE) state of Ln, which is maximized at *ca.* 830 and 940 nm for L4 and L5, respectively. Interestingly, the LE signal in each case quickly decays, leading to the formation of new spectroscopic features. For example, for the PBDB-T/L4 blend, its TA spectrum (Fig. 2a and b) obtained at 250 ps consists of three positive features, peaked at *ca.* 680, 750, and 930 nm, respectively, and negative features or GSB signals at *ca.* 600 nm that resemble the absorption spectrum of PBDB-T. As shown in Fig. 3a and b, the TA spectra of the PBDB-T/L5 blend show similar behaviors. Since the only possibility for producing a gradual loss of PBDB-T absorbance in the current case is that its redox state is changed through a HT process, the corresponding GSB signal in the 600 nm region therefore can be attributed to the formation of a PBDB-T radical cation (PBDB-T˙^+^), which leads to depletion of the ground-state PBDB-T molecules. In other words, direct photoexcitation of the NFA in these D/A blends can result in CS, a phenomenon that has also been observed in other studies.^18,21,22^

**Fig. 4:**
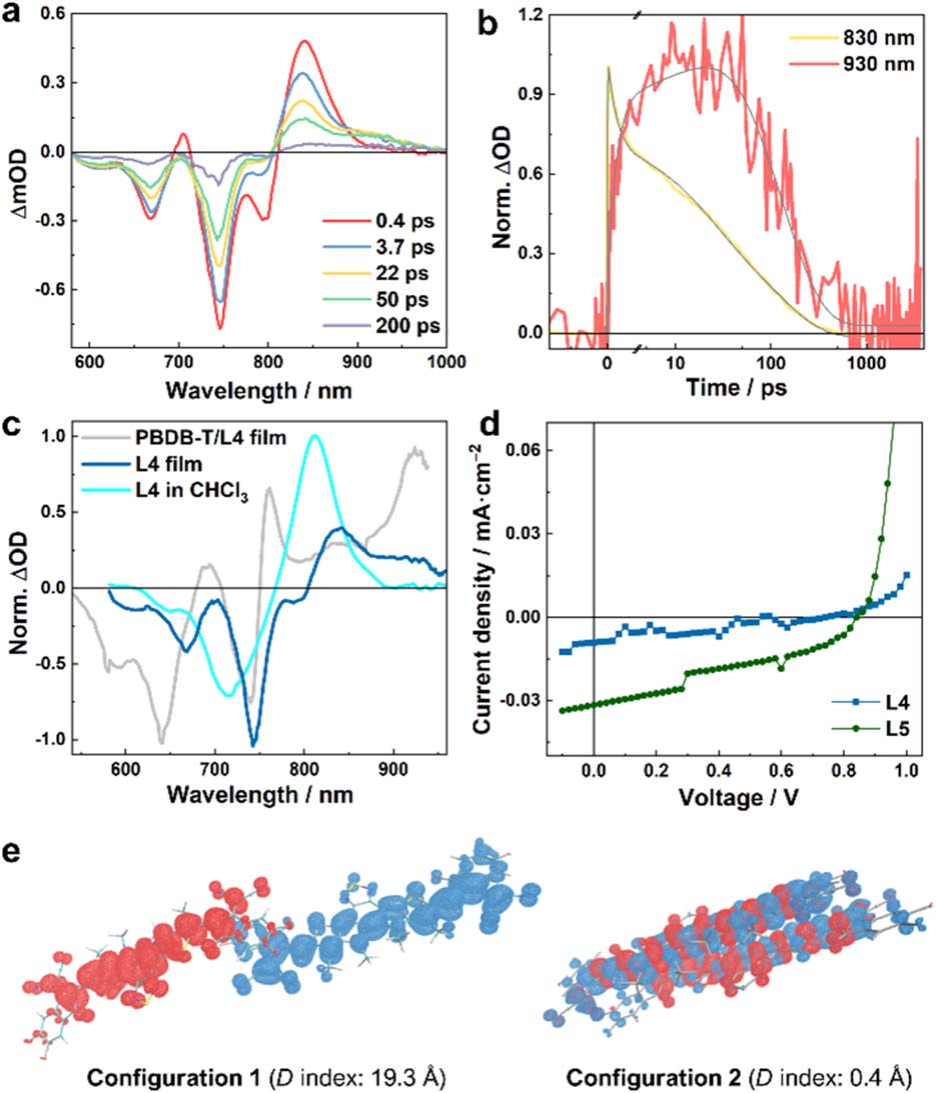
(a) TA spectra of the L4 film obtained with an excitation wavelength of 800 nm and at different time delays, as indicated, and (b) the corresponding normalized TA kinetic traces at 830 and 930 nm, respectively. (c) Normalized TA spectra at a time delay of 50 ps obtained for different samples, as indicated; the exciton wavelength for the film samples was 800 nm, whereas for the solution sample it was 700 nm. (d) *J–V* curves of neat L4- and L5-based devices. (e) Hole–electron analysis of the lowest excited states of L4 dimers in different stacking configurations. Red and blue regions denote the hole and electron distributions, respectively (isovalue = 0.0003). The centroid distances between the hole and electron (*D* index)^37^ are also indicated.

To provide further quantitative information, we globally analyzed the TA kinetics of the PBDB-T/L4 blend at key representative wavelengths: 600, 830, and 930 nm. As shown in Fig. 2c and Table 1, these kinetic traces can be globally fit by using a function consisting of an instantaneous component, an offset, and three exponentials with the following time constants: 4.4, 30.2, and 412 ps. Judging from the signs of the pre-exponential factors, it is evident that the signal at 830 nm is kinetically linked to those at 600 and 930 nm through the 4.4 ps and 30.2 ps components, as the former (latter) decays (rise) biexponentially with these two time constants. Similarly, the instantaneous components may also follow the same kinetic relationship; however, as the ground state of L4 has a nonnegligible absorbance at 600 nm and the absorption spectrum of its LE state is likely to extend to 930 nm, it is difficult to determine the exact contribution of the CS event to these components. Based on these kinetic results, it can be argued that the TA spectra obtained at a time delay of longer than *ca.* 200 ps should be composed of (at least) four spectral features: two negative ones corresponding to the absorption spectra of PBDB-T and L4 in the blend and two positive ones arising from the resultant PBDB-T radical cation (PBDB-T˙^+^) and L4 radical anion (L4˙^−^). Indeed, as shown in Fig. 2b, the TA spectrum obtained at a time delay of 250 ps clearly matches these characteristics and indicates that one positive band is peaked at *ca.* 930 nm, and another is located in the wavelength range of *ca.* 660–780 nm. While the 930 nm band most likely corresponds to the absorption spectrum of L4˙^−^ and the latter to PBDB-T˙^+^, we further validated this assignment by measuring the TA spectra of a neat L4 film and solution (in CHCl_3_) samples. As shown in Fig. 4a, c and S3a†, the corresponding results clearly indicate that the 930 nm band is present (absence) in the TA spectra obtained with the film (solution) sample. Therefore, these results not only corroborate the above assignment but also indicate that photoexcitation can induce CS in L4 film samples without the presence of an external electron donor (see further discussion below), a phenomenon that has also been suggested to occur in neat Y6 samples.^18,20^ Unlike in the current case, however, due to strong spectral overlap in the Y6 systems, a characteristic absorption feature of Y6˙^−^ cannot be directly discerned.^18,20,22^ Moreover, the fact that the formation kinetics of PBDB-T˙^+^ and L4˙^−^ are correlated with the decay kinetics of the LE state of L4 indicates that the underlying CS process is highly efficient with near-unity yield.

**Table 1:** Parameters from the global fits of the TA data of the PBDB/L4 blend film at selected probe wavelengths (*λ*_probe_) representing important spectral features with 800 nm excitation

*λ*_probe_ (nm)	*A*_1_	*τ*_1_ (ps)	*A*_2_	*τ*_2_ (ps)	*A*_3_	*τ*_3_ (ps)	Offset
600	0.01	4.4	0.04	30.2	−0.02	412	−0.06
830	0.05	4.4	0.17	30.2	0.01	412	0.02
930	−0.01	4.4	−0.12	30.2	0.05	412	0.12

The results obtained from the above kinetic analysis indicate that the CS process originated from the LE state of L4 in the blend and involves two kinetic events (or possibly with an additional unresolved ultrafast component), although the relative amplitudes of the 4.4 ps components are significantly smaller than that of the 30.2 ps component. While the fast component is typically attributed to a HT process occurring at or near the D/A interfaces,^22,33^ the interpretation of the slow components vary. For example, Natsuda *et al.*^22^ observed a TA kinetic event of *ca.* 10 ps when Y6 was photoexcited in the PM6/Y6 blend and they attributed it to spatial dissociation of the photo-generated charges driven by downhill energy relaxation, based on observation that the Y6 GSB signal shifts to longer wavelengths during this time period. On the other hand, Zhu and coworkers^33^ observed a TA kinetic event of *ca.* 100 ps when IT-4F was photoexcited in the PM6/IT-4F blend and they attributed it to a diffusion-mediated HT process. Since the TA spectra obtained with the PBDB-T/L4 blend do not show a redshift of the GSB band of the acceptor as observed in the Y6 blends^22^ (Fig. 2a and b), it seems reasonable to assume that the 30.2 ps component is attributed to CS processes following exciton diffusion to D/A interfaces. However, as will be discussed below, such an exciton diffusion process appears to be absent when the sample is excited with an excitation wavelength of 550 nm, where the sample absorbance is dominated by that of the donor, PBDB-T.

Furthermore, the signs of the 412 ps and the long-lived components indicate that they report kinetic events that lead to ground state recovery through charge recombination. This indicates that there are two types of charges in the blend; the 412 ps component is likely to correspond to the recombination of more locally bound anion–cation (electron–hole) pairs, whereas the long-lived component corresponds to those of more spatially separated pairs. This may come as a result of the heterogeneity of the local acceptor density in the blend, as has been suggested in polymer/fullerene blends.^34^ However, the fraction of the 412 ps component is relatively small (*ca.* 30%) and, hence, is not expected to play a dominant role in dictating the overall efficiency of the corresponding solar cell. Nevertheless, this finding suggests that the blend morphology is an important factor for consideration in practice, a notion that has also been emphasized in previous studies.^35^

As shown in Fig. 2d and e, except for the GSB signal of PBDB-T which appears instantaneously, the overall shapes of the TA spectra of the PBDB-T/L4 blend obtained with 550 nm excitation are similar to those measured with 800 nm excitation, especially at long time delays. For example, the TA spectra at 250 ps obtained in both cases are almost identical, indicating that both excitations produce the same products (*i.e.*, PBDB-T˙^+^ and L4˙^−^) at this time delay. However, the temporal evolution of these spectra exhibits more intricate behaviors at early time delays. First, by comparing with the TA spectra of a pure PBDB-T film (Fig. S4†), it can be concluded that a fraction of the GSB signal of L4 at 735 nm, and the positive signals in the range of *ca.* 660–780 nm assignable to PBDB-T˙^+^ are developed within the response time of the instrument (*ca.* 0.2 ps); this indicates that a fraction of the excited-state PBDB-T population is engaged in an ultrafast electron transfer (ET) process to L4 in less than *ca.* 0.2 ps. Second, the induced absorption feature ascribed to the LE state of L4 at *ca.* 830 nm appears at early time delays, indicating that a fraction of the excited-state PBDB-T population is also engaged in an energy transfer process to L4. Third, the spectral feature at *ca.* 830 nm decays to an undeletable level after 250 ps, whereas at this delay time the positive peak at *ca.* 930 nm reaches approximately its maximum, indicating that, as observed with 800 nm excitation, the fate of the excited state of L4 is to generate PBDB-T˙^+^ and L4˙^−^ through a HT process.

As done in the case of 800 nm excitation, we further globally analyzed the 550 nm excitation TA kinetics at key probing wavelengths: 590, 735, 830 and 930 nm. Similarly, as shown in Fig. 2f and Table 2, these kinetics traces can be globally fitted by using a function consisting of an instantaneous component, and three exponentials with the following time constants: 0.8, 15.1, and 520 ps, as well as an offset. Clearly, the 0.8 ps component corresponds to the energy transfer process from the excited state of PBDB-T to L4, because it leads to an increase in the signal at 830 nm (due to the formation of the LE state of L4), a decrease in the signal at 590 nm (due to ground state recovery of PBDB-T upon excited-state energy transfer), and an increase in the signal at 735 nm (due to ground state bleaching of L4 upon the formation of the LE state of L4). In addition, a fraction of the signal at 830 nm increases within the instrument response time, indicating that an ultrafast energy transfer process also occurs in the blend. On the other hand, the 15.1 ps component can be attributed to the HT process from the excited state of L4 to PBDB-T, because it leads to a decrease (increase) in the signal at 830 nm (930 nm). Furthermore, the fact that the overall decay kinetics of the system consist of two components (*i.e.*, the 520 ps and the unresolved longer decay components) indicates that there are at least two types of charges in the blend that recombine on different timescales, most likely corresponding to bound and separated electron–hole pairs, similar to those discussed above.

**Table 2:** Parameters from the global fits of the TA data of the PBDB/L4 blend film at selected probe wavelengths (*λ*_probe_) representing important spectral features with 550 nm excitation

*λ*_probe_ (nm)	*A*_1_	*τ*_1_ (ps)	*A*_2_	*τ*_2_ (ps)	*A*_3_	*τ*_3_ (ps)	Offset
590	−0.08	0.8	0.03	15.1	−0.05	520	−0.13
735	0.22	0.8	−0.28	15.1	0.01	520	−0.12
830	−0.08	0.8	0.15	15.1	0.03	520	0.06
930	−0.03	0.8	−0.10	15.1	0.05	520	0.17

The kinetic results obtained with 550 nm excitation indicate that there are two decoupled CS pathways when PBDB-T is photoexcited in the PBDB-T/L4 blend, one involving a direct (and ultrafast) ET process from D to A, and another one involving a rapid energy transfer process from D to A first, followed by a HT from A to D. Here, the timescale of the latter process (*ca.* 15 ps) is slower than the interfacial HT (*ca.* 5 ps) but faster than the diffusion-coupled HT (*ca.* 30 ps) observed with 800 nm excitation. As energy transfer is fundamentally a near-field nonradiative process with either a sixth-power distance dependence (Förster mechanism) or exponential distance dependence (Dexter mechanism), under current circumstances it can only efficiently occur at the D/A interfaces. Therefore, here it appears unlikely that the CS state formed in *ca.* 15 ps is due to a diffusion-mediated HT process as no exciton diffusion to the interfaces is required in such a scenario. In addition, the fact that a 0.8 ps energy transfer process can occur with a considerable yield when a faster ET process (<0.2 ps) is also present suggests that two (or more) distinctively different D/A interfaces exist in the blend, with one being predisposed to ultrafast ET/HT on timescales from sub-picoseconds to a few picoseconds, and another being more efficient for interfacial energy transfer.

Similar analysis can be applied to the TA results of the PBDB-T/L5 blend. While in this case the absorption spectra of the LE and CS states cannot be isolated due to spectral overlap in the range of *ca.* 860–980 nm (Fig. 3), the underlying decay kinetics are expected to be similar to those of the PBDB-T/L4 blend. Indeed, as shown in Fig. 3c, f and Tables S1 and S2†, the representative TA kinetics at 630, 820, and 930 nm can be globally fit by using a function consisting of an instantaneous component, three exponential terms, and a long-lived component. For kinetic traces obtained with 800 nm excitation, the three exponential time constants are 1.6, 13.6, and 291 ps, respectively, whereas for kinetic traces obtained with 550 nm excitation, the three exponential time constants are 0.2, 12.0, and 548 ps, respectively. Based on the rationales used in the assignment of the kinetic components of the PBDB-T/L4 blend, for results obtained with 800 nm excitation, we can attribute the 1.6 ps and 13.6 ps components to CS processes *via* the mechanism of HT, and attribute the 291 ps and the long-lived components to charge recombination. Similarly, for results obtained with 550 nm excitation, the 548 ps and the long-lived components can be assigned to charge recombination, while the 0.2 ps component should mainly correspond to an energy transfer process, as on this timescale the kinetics at 630 nm (bleaching of ground-state PBDB-T) is dominated by a decay and the kinetics at 820 nm (bleaching of ground-state L5) is dominated by an increase; the 12.0 ps component can be ascribed to a HT process following the energy transfer event. Interestingly, the CS events in the L5 blend are faster than those in the L4 blend, which may contribute to the higher PCE of L5-based devices.^32^

As discussed above, photoexcitation of the neat film sample consisting of only L4 molecules can produce measurable signals at *ca.* 930 nm that correspond to the absorption of L4˙^−^ (Fig. 4a). As shown in Fig. 4b and Table S3†, a quantitative assessment of the photoinduced kinetics indicates that this signal increases in a three-exponential manner, as observed in the blend samples, including an instantaneous component and two slower components with time constants of *ca.* 2.3 and 30.1 ps. However, the photoinduced signals at different probing wavelengths all decay with an apparent time constant of *ca.* 127 ps, which suggests that, unlike in the case of the blend samples, the photoinduced charges in the film are more locally bound and inclined to recombine to reform the LE state (or singlet excitons). This finding is consistent with the theoretical work by Ji *et al.*,^36^ which highlighted the role of D/A interfaces in impeding charge recombination. For the neat L5 film, we could not directly observe the absorption spectrum of L5˙^−^ (Fig. S5†), due to the aforementioned spectral overlap. Nevertheless, the triphasic spectral evolution (Table S4†), with time constants of 0.6, 12.7, and 127 ps, respectively, at the wavelengths corresponding to the GSB of L5 and where both LE and CS states absorb, indicates that CS events can also occur in the neat film of L5. Similar to those of L4, the 0.6 ps and 12.7 ps components should correspond to the CS (which is considerably faster than the 2.3 and 30.1 ps of the L4 film), while the 127 ps component corresponds to the charge recombination. Faster CS in the L5 film compared to the L4 film also corroborates the fact that the PBDB-T/L5 blend exhibits faster CS compared to the PBDB-T/L4 blend when the acceptors are photoexcited using an excitation wavelength of 800 nm (see above).

To provide further evidence that photoexcitation of individual L4 and L5 films can lead to CS, we measured the current density–voltage (*J*–*V*) curve of solar cells made from either pure L4 or L5. As shown in Fig. 4d and Table S5†, such devices can indeed produce photocurrent with a PCE of *ca.* 0.003% (0.011%) for L4 (L5). Single component Y6 devices have been shown to exhibit similar behavior, and quantum chemical calculations on molecular pairs based on the Y6 crystal structure suggested distinct polarization environments created by specific packing geometries of Y6 molecules as the origin of charge formation in a neat Y6 system.^20^ As there is no crystal structure of L4 or L5 available, we performed MD simulations to access molecular geometries of L4 and L5 neat films, and TDDFT calculations to investigate the excited states of the dimeric L4 and L5 molecular pairs. For both L4 and L5, dimers with multiple packing configurations were identified, *e.g.*, with compete cofacial π-stacked configurations, or different degrees of slipped stacking patterns, similar to those obtained in the MD simulations of Y6.^24,25^ The representative dimeric configurations were further subjected to DFT structure optimizations and TDDFT excited-state calculations; hole–electron analysis^37,38^ of the results obtained from TDDFT calculations reveals that for different dimeric configurations, the lowest excited states exhibit different LE/CS characteristics. The lowest excited states of dimers with only small parts (the terminal groups) of the monomers being overlaid (configuration 1 in Fig. 4e and S6†) are characterized as a well-defined charge-transferred state with the negative and positive charges separated by long distances (up to *ca.* 20 Å). By contrast, the dimers with a less slipped stacking configuration exhibit less efficient CS where the lowest excited state is essentially a LE state that involves both monomers (configuration 2 in Fig. 4e and configurations 2 and 3 in Fig. S6†). Taken together, these computational results suggest that in NFA neat films, where it is known that domains of different sizes and molecular-alignments exist,^35^ microscopic heterogeneity of molecular conformations can provide a nonnegligible driving force to allow the CS to occur, which, in turn, implies that the differences in morphologies and crystallinities of the NFA aggregates may play a role in the overall CS efficiency of the OPV system. This notion is consistent with our experimental observations and a previous study,^18^ which indicated that, because of the presence of D/A intermixed phases and the presence of NFA aggregates in the blend,^24,39^ the intermolecular charge transfer excited state in the NFA moiety may serve as an onset of CS in the blends in addition to the interfacial CS.

## Conclusions

In summary, we carry out ultrafast transient absorption spectroscopic studies on PBDB-T/L4 and PBDB-T/L5 blends as well on neat L4 and L5 films. We demonstrate that for the PBDB-T/L4 sample, the spectral features characterizing the LE state, PBDB-T˙^+^, and L4˙^−^ can be explicitly identified, thus allowing us to assess the CS kinetics through all possible channels. Specifically, we find that (1) when the acceptor (*i.e.*, L4) is selectively excited, CS can take place through two HT processes, with time constants of 4.4 and 30.2 ps, respectively and (2) when the donor (*i.e.*, PBDB-T) is selectively excited, CS can take place through either an ultrafast ET process (<0.2 ps) or an energy transfer-induced HT process with a time constant of 15.1 ps. For the PBDB-T/L5 blend, while the TA spectra are less spectroscopically distinguishable, a global kinetic analysis suggests that its CS mechanism is identical to that of the PBDB-T/L blend although the underlying kinetics are faster. Furthermore, we spectroscopically confirm that photoinduced CS can be achieved even in the neat films of L4 and L5 without the presence of additional electron donors, which is in agreement with the notion that CS can occur in OPV constructs even when the intrinsic driving force is small.^11,23^ We further apply DFT and TDDFT calculations to examine the formation and delocalization of excitons in the neat films of Ln from a theoretical standpoint, which confirms the CS in the excited states of L4 and L5 dimers. Taken together, we believe that our results provide further insights into the CS mechanisms in NFA blends, and provide direct spectroscopic evidence to support the notion that OPV devices do not require a D/A interface to dissociate excitons into charges.

## Author contributions

Wenkai Zhang and Feng Gai: conceptualization and supervision; Guangliu Ran, Bo Zhuang and Feng Gai: measurements and data analysis; Hao Lu, Yahui Liu and Zhishan Bo: synthesis and organic cell fabrication; Bo Zhuang and Jiulong Huang: theoretical calculation; Guangliu Ran, Bo Zhuang and Feng Gai: investigation and writing the original draft; Feng Gai and Wenkai Zhang: review and editing.

## Conflicts of interest

There are no conflicts to declare.

## Supplementary Material

SC-015-D4SC04072D-s001

## Data Availability

The data supporting this study are available within the article and ESI.† The raw data are available from the corresponding author upon reasonable request.
