# Supplementary material for: Electron, hole, and energy transfer dynamics in non-fullerene small-molecule acceptors
Source: Chem Sci. 2024 Sep 18;15(39):16079–85. doi: 10.1039/d4sc04072d (PMC11417900; doi:10.1039/d4sc04072d)
Supplement: SC-015-D4SC04072D-s001 [file SC-015-D4SC04072D-s001.pdf]

Supplementary Information (SI) for Chemical Science.
This journal is © The Royal Society of Chemistry 2024

Supplementary Information

Electron, hole, and energy transfer dynamics in non-fullerene small-molecule acceptors

Guangliu Ran,‡[ae] Bo Zhuang,‡[b] Jiulong Huang,[b] Hao Lu,[c] Yahui Liu,[d] Zhishan Bo,[d] Feng Gai,*[b] and Wenkai Zhang*[ae]

[a] *School of Physics and Astronomy, Applied Optics Beijing Area Major Laboratory, Center for Advanced Quantum Studies, Beijing Normal University, Beijing 100875, China.*

[b] *Beijing National Laboratory for Molecular Sciences, College of Chemistry and Molecular Engineering, Peking University, Beijing 100871, China.*

[c] *College of Materials Science and Engineering, Qingdao University, Qingdao, Shandong 266071, China*

[d] *College of Textiles and Clothing, State Key Laboratory of Bio-fibers and Eco-textiles, Qingdao University, Qingdao, Shandong 266071, China*

[e] *Key Laboratory of Multiscale Spin Physics, Ministry of Education, Beijing Normal University, Beijing 100875, China*

‡ Guangliu Ran and Bo Zhuang contribute equally to this work.

* Corresponding author. *E-mail:* fgai@pku.edu.cn; wkzhang@bnu.edu.cn.

Sample preparations.

To prepare the neat film samples, the acceptor molecules were dissolved in a mixed solvent (chloroform:chlorobenzene = 0.93:0.07, v/v) at 40 °C with a concentration of ~15 mg/mL, which was stirred for 20 min to make sure the solid was fully dissolved. Then the neat films were prepared by spin-coating (1400 rpm) from the obtained solutions in a N_2-filled glove box for 30 s. For the blend films, a mixture of PBDB-T and acceptor molecules (PBDB-T:acceptor = 1:1, w/w) were dissolved in chlorobenzene at 100 °C and stirred for 20 min to make sure the solid was fully dissolved. The concentration of PBDB-T was set as ~4 mg/mL. The blend films were prepared by spin-coating (1400 rpm) from the mixture solutions in a N_2-filled glove box for 30 s, and then annealed at 150 °C for 5 min.

Spectroscopic measurements.

For all the samples, steady-state absorption spectra were recorded with an Agilent Technologies Cary 60 UV−Vis spectrophotometer. For femtosecond transient absorption measurements, a Yb:KGW laser (1030 nm, 54 kHz, Light Conversion) is split into two fundamental light beams. One of the light beams is transferred to the optical parametric amplifier (Orpheus, Light Conversion) to generate a high-intensity pulse of a specific wavelength as the pump beam. At the same time, the other is focused on a 5 mm sapphire to generate low-intensity continuum light, employed as the probe beam. The pump and probe beams spatially were set at the magic angle (54.7°) and spatially overlapped at the sample. The time delays between pump and probe beams were achieved using a delay stage monitor, and the transmitted probe light is collected using a charge-coupled device. The pump fluence was kept at ca.2 $\mu J/cm^2$, unless indicated otherwise. For the film samples, the transient absorption experiment was done six times on several spots of the film for each sample, of which the average was taken. Upon completion, no sample degradation was detected. Global analysis of the time-resolved spectroscopic data was performed using the Glotaran program.[1]

Computer simulations.

MD simulations were performed using the NAMD program (version 3.0b3).[2] Force field parameters of L4 and L5 were generated by the CGenFF program (interface: version 1.0.0; force field: version 3.0.1)[3,4] and additional improper dihedral restraints were added to re-enforce

planarity of the molecules. 200 molecules of L4 and L5 molecules were randomly placed in an cubic simulation box at low density (~0.1 g/cm^3) using the PACKMOL package (version 18.169).[5] Periodic boundary conditions were assumed and the integration time step was set at 0.5 fs. A 30 ns of NPT-MD was performed at 650 K and 1 atm, then the systems were cooled at 1 atm from 650 K to 300 K at a rate of 10 K/ns followed by another 30 ns of NPT-MD at 300 K and 1 atm.[6] Representative dimers of L4 or L5 selected from the last 10 ns of the MD simulations were further subjected to DFT and TDDFT calculation, using the ORCA package (version 5.0.4)[7] DFT geometry optimizations were performed with the r^{2}SCAN-3c composite method[8] implemented in ORCA, and TDDFT calculations were performed the cam-B3LYP[9]/def-SVP levels based on the DFT-optimized geometries. In these calculations a dielectric constant ε=3.5 in CPCM implicit solvent model was used to represent the effects of surrounding NFA molecules,[10] and the long alkyl and alkoxy sidechains were reduced to methyl and methoxy groups, respectively, for simplicity. Hole and electron analysis of the excited states were performed using the Multiwfn program (version 3.8).[11] Visualization of the results were carried out with the aid of the VMD programs (version 1.9.4a53).[12]

Fabrication and characterization of the solar cells.

Pre-patterned ITO-coated glass substrates were cleaned with detergent and ultrasonicated in deionized water, acetone and isopropanol for 20 min each and subsequently dried in an oven overnight. Device was fabricated with conventional device structure of ITO/2PACz/Acceptor/PNDIT-F3N/Ag. Ethanol solution of 2PACz (Purchased from Tokyo Chemical Industry) was spin-cast onto the ITO surface at 6000 rpm for 30 s (the thickness is about 8 nm), and baked at 60 °C for 1 min in air. Dissolve the acceptor in solution (chloroform:chlorobenzene = 0.93:0.07) at 40 ° C and stir for 20 min to make sure the solid was fully dissolved. The concentration of acceptors was set as 15 mg/mL. The active layers were spin-coated from chloroform solutions in a N_2-filled glove box. After the layers were fully dried, the methanol solution of PNDIT-F3N (0.5 mg mL^{-1}) was spin-cast on the BHJ layer at 2000 rpm. Finally, a 100 nm Ag layer was thermally deposited at a vacuum pressure below 10^{-7} Torr. Photovoltaic cells were fabricated on the substrate with an effective area of 0.04 cm^2. The *J–V* curves of photocurrents were recorded in glove box at ~25 °C using an instrument from Enli Technology Ltd., Taiwan (SS-F53A) under AM 1.5G illumination (AAA class solar simulator, with an intensity of 100 mW cm^{-2} calibrated with a standard single crystal Si photovoltaic cell).

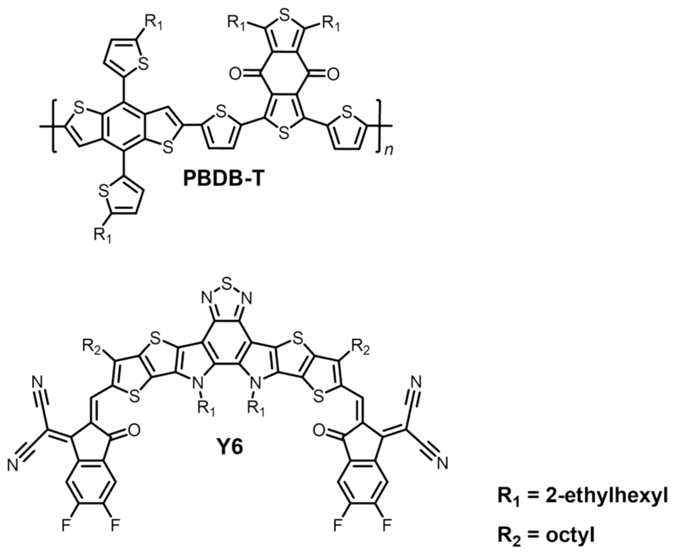

Fig. S1 Chemical structure of PBDB-T and Y6, as indicated.

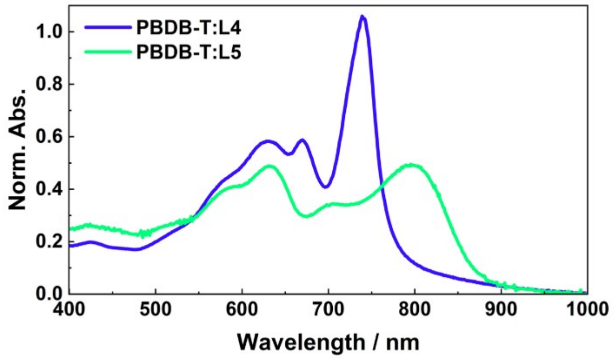

Fig. S2 UV-visible absorption spectra of PBDB-T/Ln blend films (PBDB-T:Ln = 1:1, w/w).

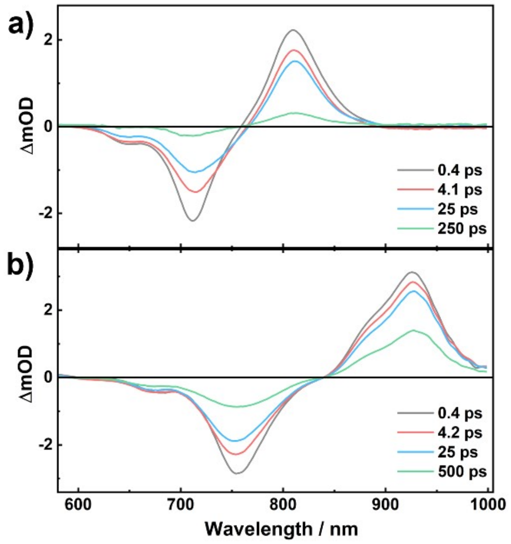

Fig. S3 TA spectra of the $CHCl_3$ solutions of L4 (a, pumped at 700 nm) and L5 (b, pumped at 760 nm) in a concentration of ca. 0.02 mg/mL, recorded at selected time delays.

Table S1 Parameters from the global fits of the TA data of the L5 blend film at selected wavelengths representing important spectral features with 800-nm excitation.

λ_{probe} (nm)	A_1	τ_1 (ps)	A_2	τ_2 (ps)	A_3	τ_3 (ps)	Offset
630	0.06	1.6	0.07	13.6	−0.02	291	−0.14
820	−0.22	1.6	−0.22	13.6	−0.02	291	−0.02
930	0.15	1.6	0.11	13.6	0.03	291	0.06

Table S2 Parameters from the global fits of the TA data of the L5 blend film at selected wavelengths representing important spectral features with 550-nm excitation.

λ_{probe} (nm)	A_1	τ_1 (ps)	A_2	τ_2 (ps)	A_3	τ_3 (ps)	offset
630 nm	−0.51	0.2	0.02	12.0	−0.04	548	−0.37
820 nm	0.33	0.2	−0.24	12.0	−0.01	548	−0.06
930 nm	−0.18	0.2	0.15	12.0	0.06	548	0.13

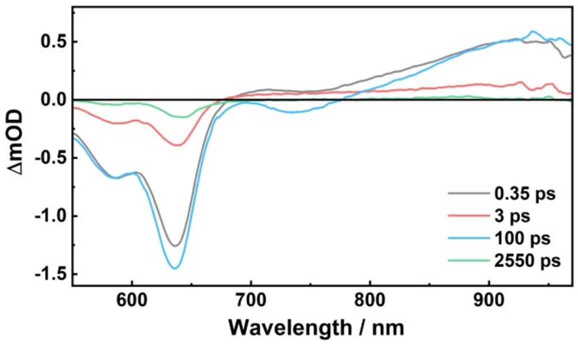

Fig. S4 TA spectra of the pure PBDB-T film measured at selected time delays, with 500-nm excitation.

Table S3 Parameters from the global fits of the TA data of the L4 neat film at selected wavelengths representing important spectral features with 800-nm excitation.

λ_{probe} (nm)	A_1	τ_1 (ps)	A_2	τ_2 (ps)	A_3	τ_3 (ps)
765 nm	−0.09	2.3	−0.09	30.1	−0.10	126
830 nm	0.12	2.3	0.16	30.1	0.15	126
930 nm	−0.05	2.3	−0.02	30.1	0.09	126

Table S4. Parameters from the global fits of the TA data of the L5 neat film at selected wavelengths representing important spectral features with 800-nm excitation.

λ_{probe} (nm)	A_1	τ_1 (ps)	A_2	τ_2 (ps)	A_3	τ_3 (ps)
820 nm	−0.16	0.6	−0.16	12.7	−0.26	127
930 nm	0.06	0.6	0.16	12.7	0.15	127

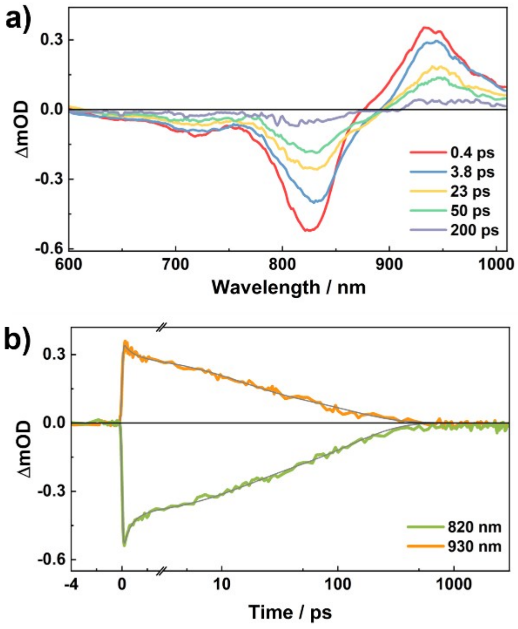

Fig. S5 TA spectra (a) and kinetics (b) of the neat L5 film measured at selected time delays with 800-nm excitation.

Table S5. Photovoltaic performance of neat L4- and L5-based devices.

Devices	V_{oc} (V)	J_{sc} (mA cm^{-2})	FF (%)	PCE (%)
L4	0.73	0.009	40.66	0.003 (0.002^{a})
L5	0.84	0.032	41.76	0.011 (0.008^{a})

^{a}Averaged data are calculated over 5 independent devices.

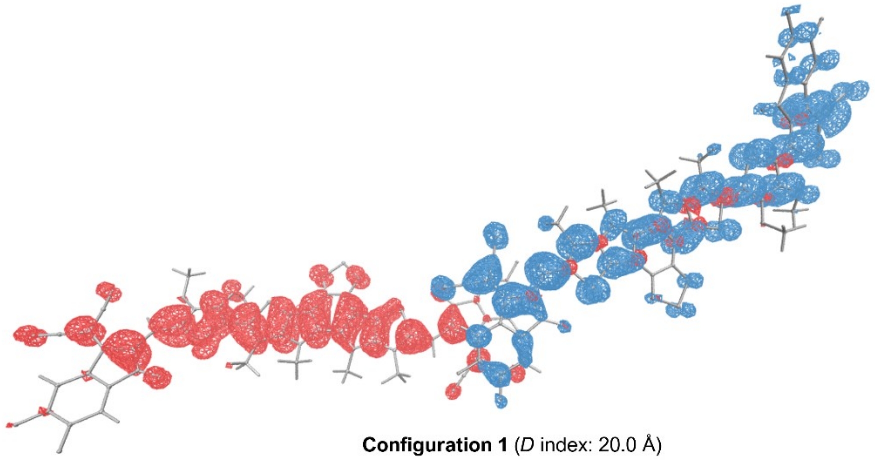

Configuration 1 (*D* index: 20.0 Å)

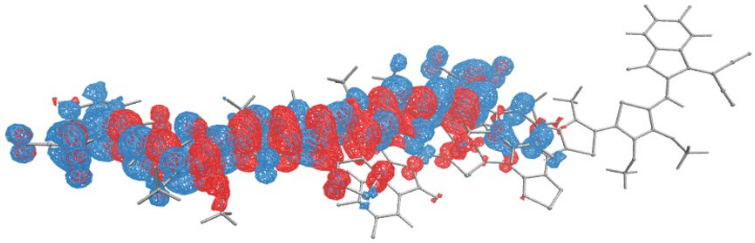

Configuration 2 (*D* index: 1.0 Å)

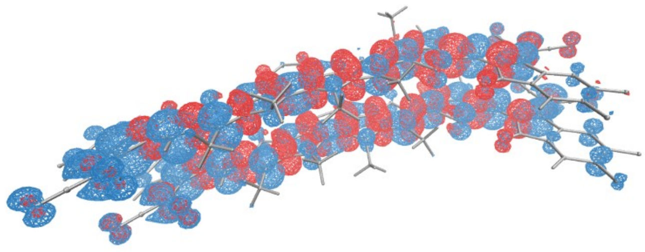

Configuration 3 (*D* index: 1.2 Å)

Fig. S6 Hole–electron analysis of the lowest excited states of L5 dimers in different stacking configurations. Red and blue regions denote the hole and electron distributions, respectively (isovalue = 0.0003). The centroid distances between the hole and electron (*D* index)[11] are also indicated.

Supporting references

(1) Snellenburg, J. J.; Laptenok, S. P.; Seger, R.; Mullen, K. M.; van Stokkum, I. H. M. Glotaran: A Java-Based Graphical User Interface for the R Package TIMP. *J. Stat. Softw.* **2012**, *49* (3).

(2) Phillips, J. C.; Braun, R.; Wang, W.; Gumbart, J.; Tajkhorshid, E.; Villa, E.; Chipot, C.; Skeel, R. D.; Kalé, L.; Schulten, K. Scalable Molecular Dynamics with NAMD. *J. Comput. Chem.* **2005**, *26* (16), 1781–1802.

(3) Vanommeslaeghe, K.; MacKerell, A. D. Automation of the CHARMM General Force Field (CGenFF) I: Bond Perception and Atom Typing. *J. Chem. Inf. Model.* **2012**, *52* (12), 3144–3154.

(4) Vanommeslaeghe, K.; Raman, E. P.; MacKerell, A. D. Automation of the CHARMM General Force Field (CGenFF) II: Assignment of Bonded Parameters and Partial Atomic Charges. *J. Chem. Inf. Model.* **2012**, *52* (12), 3155–3168.

(5) Martinez, L.; Andrade, R.; Birgin, E. G.; Martínez, J. M. PACKMOL: A Package for Building Initial Configurations for Molecular Dynamics Simulations. *J. Comput. Chem.* **2009**, *30* (13), 2157–2164.

(6) Kupgan, G.; Chen, X. K.; Brédas, J. L. Molecular Packing of Non-Fullerene Acceptors for Organic Solar Cells: Distinctive Local Morphology in Y6 vs. ITIC Derivatives. *Mater. Today Adv.* **2021**, *11*, 100154.

(7) Neese, F. The ORCA Program System. *Wiley Interdiscip. Rev. Comput. Mol. Sci.* **2012**, *2* (1), 73–78.

(8) Ehlert, S.; Huniar, U.; Ning, J.; Furness, J. W.; Sun, J.; Kaplan, A. D.; Perdew, J. P.; Brandenburg, J. G. R2SCAN-D4: Dispersion Corrected Meta-Generalized Gradient Approximation for General Chemical Applications. *J. Chem. Phys.* **2021**, *154* (6), 61101.

(9) Yanai, T.; Tew, D. P.; Handy, N. C. A New Hybrid Exchange–Correlation Functional Using the Coulomb-Attenuating Method (CAM-B3LYP). *Chem. Phys. Lett.* **2004**, *393* (1–3), 51–57.

(10) Zhang, G.; Chen, X. K.; Xiao, J.; Chow, P. C. Y.; Ren, M.; Kupgan, G.; Jiao, X.; Chan, C. C. S.; Du, X.; Xia, R.; Chen, Z.; Yuan, J.; Zhang, Y.; Zhang, S.; Liu, Y.; Zou, Y.; Yan, H.; Wong, K. S.; Coropceanu, V.; Li, N.; Brabec, C. J.; Bredas, J. L.; Yip, H. L.; Cao, Y. Delocalization of Exciton and Electron Wavefunction in Non-Fullerene Acceptor Molecules Enables Efficient Organic Solar Cells. *Nat. Commun.* **2020**, *11* (1), 1–10.

(11) Lu, T.; Chen, F. Multiwfn: A Multifunctional Wavefunction Analyzer. *J. Comput. Chem.* **2012**, *33* (5), 580–592.

(12) Humphrey, W.; Dalke, A.; Schulten, K. VMD: Visual Molecular Dynamics. *J. Mol. Graph.* **1996**, *14* (1), 33–38.
